# Anesthetic Management of a Patient With a Ruptured Heterotopic Pregnancy for Emergency Exploratory Laparotomy

**DOI:** 10.7759/cureus.16850

**Published:** 2021-08-03

**Authors:** Sameep Dhillon, Gibbs Yim, Rashmi Vandse

**Affiliations:** 1 Anesthesiology, Loma Linda University School of Medicine, Loma Linda, USA; 2 Anesthesiology, Loma Linda University Medical Center, Loma Linda, USA

**Keywords:** obstetrical hemorrhage, nonobstetrical surgery, heterotopic pregnancy, ectopic pregnancy, non-obstetrical surgery

## Abstract

Heterotopic pregnancy (HP) is an uncommon clinical condition characterized by the coexistence of intrauterine and extra-uterine pregnancies. HP is a diagnostic challenge as the typical methods for the early diagnosis of ectopic pregnancy are confounded by the simultaneous presence of intrauterine pregnancy (IUP). Ruptured HP is a potentially life-threatening obstetric emergency and can result in significant morbidity and mortality. Early diagnosis is the key to a favorable outcome. With the increasing number of patients undergoing artificial reproductive technology (ART), which is an important risk factor for HP, the odds of encountering HP patients are also growing. Anesthesiologists are challenged by the need to manage a bleeding obstetric patient while simultaneously ensuring the safety of the IUP. We present perioperative management of a patient with bleeding ruptured HP for emergency laparotomy who went on to have a successful twin delivery 37w3d via cesarean section.

## Introduction

Heterotopic pregnancy (HP) is a very rare and potentially life-threatening obstetric event in which there are simultaneous intrauterine and extrauterine gestations. [1-3]. The incidence of HP following spontaneous conception is around one in 30,000 [1-3], while in pregnancies attained following artificial reproductive technology (ART) the incidence has been reported to be as high as 1:100 [2-4]. Risk factors for HP include ART, ovarian induction, history of tubal surgery, intrauterine device, dilatation and curettage (DC), and history of pelvic inflammatory disease (PID) [5]. Among these, ART has been recognized as the strongest risk factor for HP. With the uprising number of ART-treated patients, the frequency of encountering HP in the perioperative setting is only expected to increase.

There is a very high risk for women with HP to present emergently after the rupture of the ectopic pregnancy. Anesthesiologists are faced with a unique situation requiring them to emergently anesthetize and resuscitate an actively hemorrhaging obstetric patient while also safeguarding the intrauterine pregnancy (IUP). While HP and its management have been well documented and discussed in obstetric literature, there is very limited data in the anesthetic literature [4-6]. Here, we present a case revolving around the anesthetic management of a patient with simultaneous dichorionic-diamniotic twin IUP and a ruptured intra-abdominal pregnancy.

## Case presentation

Our patient was a 37-year-old Gravida 4, Para 0 female who was morbidly obese (BMI 48) and had a history of endometrial polyp status post-polypectomy, chronic hypertension, Hashimoto’s thyroiditis with resulting hypothyroidism on treatment, and hiatal hernia. She underwent successful ART treatment via clomiphene and intrauterine insemination and was diagnosed with known twin IUP.

She presented to the emergency department at 12w1d with an acute onset of abdominal and shoulder pain. She was hemodynamically stable but was tachycardic. Her physical exam showed a tender distended abdomen. Hemoglobin was 9.1 g/dL, which had dropped from 12.1 g/dL three weeks prior. Serum beta-HCG was 87,000. In addition to the previously known twin IUP, an abdominal ultrasound revealed free fluid in the abdomen along with a left adnexal ectopic pregnancy. At this point, the patient was taken to the operating room for an emergent diagnostic laparoscopy.

She was premedicated with midazolam. Adequate intravenous access was obtained prior to induction. Rapid sequence induction was achieved using fentanyl, lidocaine, propofol, and succinylcholine. With optimum patient positioning and preoxygenation, the airway was secured by video laryngoscopy. The right radial artery was cannulated for hemodynamic monitoring and necessary lab work. Anesthesia was maintained with sevoflurane and rocuronium. Laparoscopy revealed an actively bleeding ruptured left fallopian tube along with an intra-abdominal pregnancy. There was significant bleeding obscuring the surgical view, hence the obstetric team converted to open laparotomy. The ectopic pregnancy was removed and left salpingectomy was performed along with evacuation of 1.5 liters of hemoperitoneum.

The patient was kept hemodynamically stable throughout the course of the surgery by volume resuscitation along with a phenylephrine infusion. She was resuscitated with two units of packed red blood cells, two units of fresh frozen plasma, 1.2 liters of crystalloid, and 0.5 liters of colloid along with 1 g of tranexamic acid (TXA). At the end of the case, neuromuscular blockade was reversed and the patient was extubated successfully. Intraoperative lung-protective strategy with recruitment maneuvers and multimodal analgesia aided in successful extubation. Fetal monitoring was appropriate for the twins in the immediate postoperative period. Postoperatively, pulmonary toilet, early mobilization, physical therapy, and venous thromboembolism prophylaxis were employed. The patient went on to have an uncomplicated postoperative recovery. Her subsequent obstetric course was unremarkable and she delivered healthy twins at 37w3d via cesarean section.

## Discussion

Despite the availability of superior diagnostic methods for early discovery and management, hemorrhage from ectopic pregnancy is still the prime cause of pregnancy-related maternal mortality in the first trimester and accounts for about 4% of all pregnancy-related deaths [7,8]. However, HP is a diagnostic challenge as the presence of IUP often obscures the results of the diagnostic tests. Serum beta-HCG levels of extrauterine pregnancy are attributed to the IUP and ultrasound often misses ectopic pregnancy when there is an accompanying IUP. In our case, the ectopic pregnancy was missed twice by prior ultrasounds performed by both the ED physicians and the formal obstetric ultrasound team. With these diagnostic challenges, there is a very high risk for women with HP to present emergently after the rupture of the ectopic pregnancy as highlighted by our case. However, studies have shown that in the hands of an experienced sonographer directed to examine for heterotopic pregnancies, the sensitivity of first trimester transvaginal ultrasound in ART-treated patients is as high as 92.4% [1]. The incidence of ART has tripled from 1996 to 2016 [9]; hence, a high index of suspicion for HP must be maintained in those with pregnancies achieved through ART.

Anesthetic and obstetric considerations

As opposed to a patient presenting with a ruptured ectopic pregnancy alone, a ruptured HP requires careful consideration of maternal-fetal physiology, pharmacodynamics, and pharmacokinetics in addition to the management of hypovolemic shock. Based on the limited evidence, the maternal and fetal outcomes are no different in general compared with regional anesthesia techniques as reported by Liu et al. in a study encompassing 49 patients [4]. However, 43 of 49 patients had conceived following ART and they had undergone regular ultrasound examination following embryo transfer, which led to early diagnosis and none of these patients had a massive hemorrhage.

For exploratory laparotomy in a ruptured HP, neuraxial anesthesia may be less prudent due to the patient’s hypovolemic state. Additionally, if the obstetricians start with diagnostic laparoscopy, neuraxial anesthesia may provide inferior surgical conditions.

The main goals of anesthetic management from the obstetric standpoint are to prevent fetal asphyxia by preserving maternal oxygenation, ventilation, and hemodynamic stability. The fetal heart tones (FHT) were checked pre- and postoperatively, which remained stable. We established large-bore intravenous access and arterial lines prior to induction for early detection and treatment of any adverse hemodynamic changes. Intra-arterial lines provided beat-to-beat fluctuations in blood pressure that can be clinically significant in an actively bleeding patient. In addition, pulse pressure variation (PPV) helped with the goal-directed fluid therapy. Due to the relative passive dependence of the uteroplacental circulation, maintaining normal maternal blood pressure is crucial. We used a phenylephrine infusion to maintain the patient’s blood pressure near her baseline. Studies have confirmed superior maternal cardiovascular stability and better neonatal acid-base status when phenylephrine was utilized for treating maternal hypotension [10,11].

In addition to blood products and IVF, we administered 1 g TXA at the beginning of the case. The safety and efficacy of TXA in reducing death due to postpartum hemorrhage have been established by the WOMAN trial [12]. TXA is also commonly used in the treatment of several inherited bleeding disorders during pregnancy. Even though TXA crosses the placenta, the available evidence does not suggest any harm to the fetus or neonates [13].

There is no convincing evidence that any specific anesthetic drug has any teratogenic effects in humans at clinically used doses [14]. Based on the evidence from large outcome studies on women who underwent surgery during pregnancy [15,16], there is no increase in congenital anomalies among their children. However, there is some evidence of the rise in the rate of abortions, preterm deliveries, and growth restriction for reasons thought to be due to the requirement for surgery and underlying conditions but not due to anesthetic drugs [15,16].

Outcome of HP

A retrospective review of 56 patients with HP showed a favorable outcome for IUP following surgical treatment with an 82.14% live birth rate. [17]. However, HP was diagnosed early in the majority of these patients. The mean gestational age was 50.61 ± 8.18 days at the time of diagnosis. Intraoperatively, nine of them had evidence of ruptured ectopic pregnancy and only three of these patients had hemoperitoneum of more than 1,000 mL requiring blood transfusion. Tal et al. [3] reported a live birth rate of 66% in 139 women, while Gorka et al. [18] reported a live birth rate of 68.75% in 80 patients with HP.

## Conclusions

In cases of hemodynamically unstable reproductive-aged women, the classical teaching to “think ectopic” holds true even in those with known IUP. Bleeding from the ruptured ectopic pregnancy can be life threatening. Consequently, early diagnosis with rapid resuscitation and maintenance of maternal hemodynamic stability is imperative for favorable maternal and fetal outcomes. As for anesthetic management, there is no “perfect anesthetic drug” and should be tailored to the patient’s clinical presentation.
